# Abundance trade-offs and dominant taxa maintain the stability of the bacterioplankton community underlying *Microcystis* blooms

**DOI:** 10.3389/fmicb.2023.1181341

**Published:** 2023-05-19

**Authors:** Jun Chen, Tiange Zhang, Lingyan Sun, Yan Liu, Dianpeng Li, Xin Leng, Shuqing An

**Affiliations:** ^1^School of Life Science and Institute of Wetland Ecology, Nanjing University, Nanjing, China; ^2^Nanjing University Ecology Research Institute of Changshu (NJUecoRICH), Changshu, China

**Keywords:** abundance trade-offs, bacterioplankton, *Microcystis*, closed-lake management, dominant taxa

## Abstract

*Microcystis* blooms are an intractable global environmental problem that pollute water and compromise ecosystem functioning. Closed-lake management practices keep lakes free of sewage and harmful algae invasions and have succeeded in controlling local *Microcystis* blooms; however, there is little understanding of how the bacterioplankton communities associated with *Microcystis* have changed. Here, based on metagenomic sequencing, the phyla, genera, functional genes and metabolic functions of the bacterioplankton communities were compared between open lakes (underlying *Microcystis* blooms) and closed lakes (no *Microcystis* blooms). Water properties and zooplankton density were investigated and measured as factors influencing blooms. The results showed that (1) the water quality of closed lakes was improved, and the nitrogen and phosphorus concentrations were significantly reduced. (2) The stability of open vs. closed-managed lakes differed notably at the species and genus levels (*p* < 0.01), but no significant variations were identified at the phylum and functional genes levels (*p* > 0.05). (3) The relative abundance of *Microcystis* (Cyanobacteria) increased dramatically in the open lakes (proportions from 1.44 to 41.76%), whereas the relative abundance of several other dominant genera of Cyanobacteria experienced a trade-off and decreased with increasing *Microcystis* relative abundance. (4) The main functions of the bacterioplankton communities were primarily related to dominant genera of Proteobacteria and had no significant relationship with *Microcystis*. Overall, the closed-lake management practices significantly reduced nutrients and prevented *Microcystis* blooms, but the taxonomic and functional structures of bacterioplankton communities remained stable overall.

## 1. Introduction

Over the past decades, there has been a rise in the frequency and distribution of cyanobacterial blooms worldwide (Paerl, 2014; Almanza et al., 2019), and the extensive propagation and decay of algae has reduced the dissolved oxygen content of water, compromising ecosystem health (O’Boyle et al., 2016; Zhao et al., 2019). In particular, the public has become aware of *Microcystis*, a genus of Cyanobacteria synonymous with harmful algal blooms (HABs). For example, a drinking water crisis caused by toxic *Microcystis aeruginosa* led directly to 2 million people unable to safely access municipal water for more than a week in May 2007 in Wuxi, Jiangsu Province, China (Qin et al., 2010). In August 2014, more than 400,000 people were subject to a ‘do not drink advisory’ for nearly 48 h because of contamination by microcystins in Toledo, Ohio, United States (Steffen et al., 2017). Since that time, numerous investigations and experiments have analysed the organic matter and genotypic structure of *Microcystis* and cyanobacteria blooms (Chen et al., 2018), long-term bloom changes (Zhang M. et al., 2021), and environmental driving forces of blooms (Zhang J. et al., 2021). However, cyanobacterial blooms are a largely unresolved global environmental problem (Ho et al., 2019; Wilhelm et al., 2020).

Wetland construction provides essential protection and management of many small shallow lakes through sewage discharge control and aquaculture implementation to improve water quality (Zhu et al., 2014) and tourism development control to enhance biodiversity (Li et al., 2010). For instance, by building sluices to isolate lakes from rivers, small lakes in Taihu National Wetland Park were constructed to prevent periodic local cyanobacterial outbreaks (Wang et al., 2020). Similarly, in a study of closed-lake management practices, Xiao et al. (2019) studied the Donghu Lake area (Wuhan, China), which is closed off by tunnel construction, based on 16S rRNA gene sequencing and found that Cyanobacteria abundance in the closed lake area was much lower than that in open lake areas. This represents a new perspective that closed-lake management practices may be a powerful precautionary strategy for preventing HABs. In contrast, Wang et al. (2020) compared the bacterioplankton communities in 3 types of closed ponds based on 16S rRNA gene sequencing and found that slight eutrophication in closed ponds led to an increase in Cyanobacteria. To further investigate the ecological effects of closed-lake management practices, it is necessary to consider changes in both cyanobacteria and *Microcystis* (Berg et al., 2018), as well as responses to the associated planktonic microbiome (Shi et al., 2010; Pound et al., 2021).

Recently, high-throughput sequencing has been used to explore the dynamics of the bacterial community (Chen et al., 2018), also to examine the consequences of cyanobacterial blooms on both the cyanosphere and the wider bacterioplankton community. Louati et al. (2016) surveyed a recreational lake and found that changes in the composition of cyanobacterial species led to significant changes in the bacterial communities associated with bloom-forming freshwater cyanobacteria. Similarly, Shen et al. (2019) compared lakes with different nutrient states and found that the functional structure of the bacterioplankton community was significantly different from that of other communities dominated by Cyanobacteria. Liu et al. (2019) also found that the biomass of Cyanobacteria during a bloom strongly affected the community composition of microeukaryotic plankton in a reservoir. In contrast, an enclosure experiment showed that aquatic bacterial communities were resilient and therefore generally stable in the face of disturbance (Shade et al., 2011). Although these 16S rRNA gene sequencing methods are useful, they have limitations in terms of identifying species. It is necessary to implement metagenomic methods for further analysis, which are better suited for identifying both species and functional genes (Shen et al., 2019; Yancey et al., 2022).

Here, we used metagenomic analysis to compare the taxonomic and functional structure of bacterioplankton communities of open lakes (*Microcystis* blooms) and artificially closed lakes (no *Microcystis* blooms) along the Wangyu River. The study reinforced the ecological effects of closed-lake management practices on controlling nutrient loading and mitigating *Microcystis* blooms.

## 2. Methods

### 2.1. Study area and sampling

**Figure 1:**
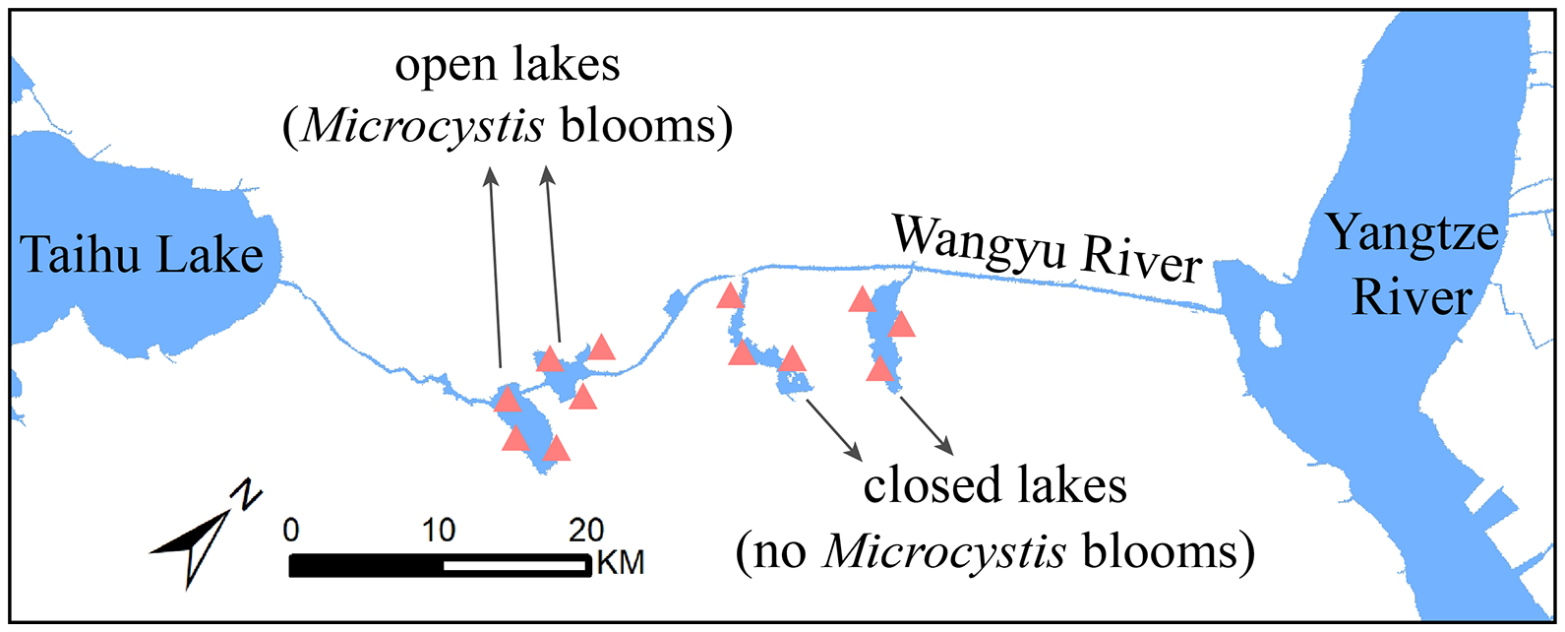
Map of sampling stations. A total of 12 stations were set up, including in open lakes (Chaohu Lake and Ehu Lake) and closed lakes (Shanghu National Wetland Park and Nanhu Provincial Wetland Park).

The Wangyu River (31°39′N, 120°38′E), a major tributary of Taihu Lake, is used to divert water from the Yangtze River. In the Taihu Basin river network, the open lakes, which are connected to the river, are subject to direct influence from the water diversion project’s navigation and fisheries efforts. On the other hand, the closed lakes were formed as a part of a wetland development initiative and are not directly connected to the river. Hence, we have selected 2 closed lakes with the same closed-lake management and 2 adjacent open lakes for our research. Sites at 3 locations away from immediate influence of large aquatic plants were selected for sampling in each lake (Figure 1).

In July 2021, cyanobacterial blooms continued to occur in the open lakes for a month but did not occur in the closed lakes. At each sampling site, surface water (<0.5 m) was collected using a 5 L water harvester. Using individual filter units (Nalgene, United States), 1 L water was passed through 47 mm, 0.22 μm hydrophilic nylon membranes (Merck Millipore, Germany), which were then separately packed into centrifuge tubes and stored on dry ice (−80°C) for metagenomic sequencing. In addition, 100 mL of surface water was collected using sterile bottles (3 replicates), and approximately 1 mL of dilute sulphuric acid was added to acidify the water to a pH < 2 and stored away from light for nutrient analysis.

Zooplankton collection involved two methods: the protozoan and rotiferan samples were placed in plastic bottles to which 1 L of surface water with 5 mL Lugol solution was added; the cladoceran and copepod samples were collected by passing 20 L of surface water through a 64 um mesh diameter plankton net and 5 mL formaldehyde solution was added for fixation.

### 2.2. Biotic and abiotic factor analysis

Temperature, oxidation–reduction potential (ORP), pH, salinity, dissolved oxygen (DO) and electrical conductivity (EC) were measured in the field with an AP-800 handheld metre (Aquaread, United Kingdom). Total phosphorus (TP), labile phosphate (labile P), total nitrogen (TN), total oxidised nitrogen (TON) and ammonium nitrogen (ammonium N) were analysed using an auto discrete analyser Cleverchem-200 (DeChem-Tech, Germany). Chemical oxygen demand (COD) was analysed using TNT-821 kits and a DR-3900 instrument (HACH, United States). The zooplankton species were identified using a 1 mL counting plate under an optical microscope at 100–400 × magnification.

### 2.3. Metagenomic analysis

Microbial community genomic DNA was extracted from the freshwater samples using the FastDNA® SPIN Kit (Omega Bio-Tek, Norcross, GA, United States) following the manufacturer’s instructions. The DNA extract was checked on a 1% agarose gel, and DNA concentration and purity were determined with a NanoDrop 2000 UV–vis spectrophotometer (Thermo Scientific, Wilmington, DE, United States; Zheng et al., 2022). On an Illumina Genome Analyser IIx, metagenome sequencing was performed and yielded >8 GB per library (>50 M reads, 150 bp paired-end reads, insert size = 500 bp). Quality trimming was performed in Fastp v0.20.0, and reads <20 bp were discarded. After filtering, we used Multiple Megahit v1.1.2 (Li et al., 2015) to assemble the metagenomics data. CD-HIT software (Fu et al., 2012) was used to cluster a nonredundant gene catalogue (90% identity, 90% coverage). The taxonomic annotation and functional analysis were performed based on the NR (Non-Redundant Protein Sequence Database) and KEGG (Kyoto Encyclopedia of Genes and Genomes), respectively.

### 2.4. Statistical analysis

The bacterioplankton community stability was evaluated by average variation degree (AVD), which is calculated using the deviation degree from the mean of the normally distributed relative abundance of species (genus, phylum or functional gene) between open and closed lakes. Lower AVD value indicates higher bacterioplankton community stability (Xun et al., 2021).

The variance inflation factor (VIF) was used to filter the autocorrelated environmental factors, and these factors were filtered several times until the VIF values corresponding to the selected environmental factors were all less than 10. Nonmetric multidimensional scaling analyses (NMDS) were used to reduce and rank species and functions to directly characterise the degree of differences between sampling stations. Distance-based redundancy analysis (db-RDA) used Bray–Curtis distance calculations to analyse the relationship between species or functions and environmental factors. The Wilcoxon rank-sum test was used to analyse the species/function differences between the two groups of samples. Correlation network analysis was calculated to construct the correlation network of species and function.

Statistical analyses were performed in R 4.0.3 and Python 3.11. Origin 2021 was used to create figures. The geographic and connectivity variables were calculated in ArcGIS 10.8.

## 3. Results

### 3.1. Diversity and stability of bacterioplankton communities

**Figure 2:**
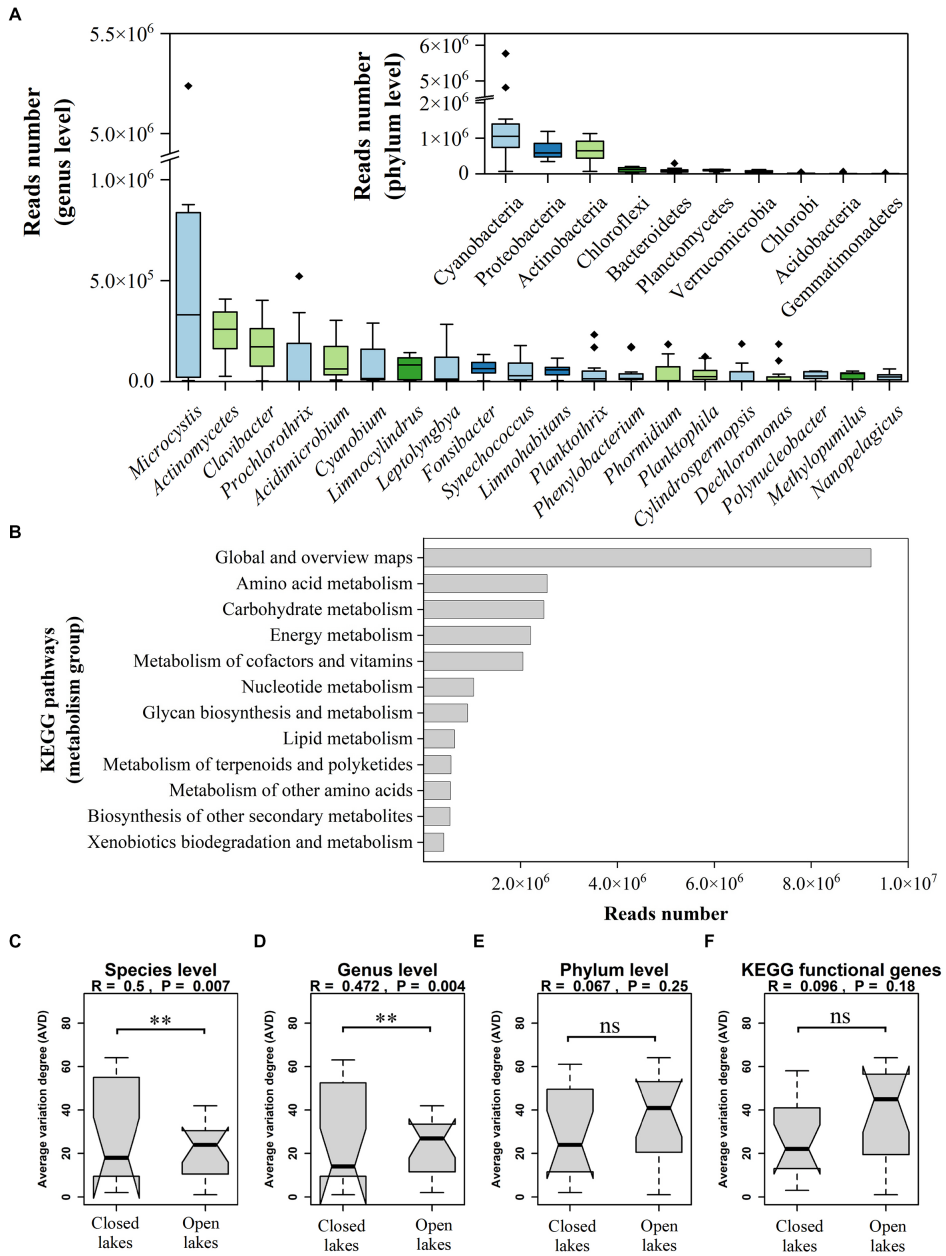
**(A)** Genus and phylum relative abundance variation box plot for the most abundant genera and phyla as determined by read relative abundance. Genera are coloured by their respective phylum. **(B)** The relative abundance of metabolic functional genes in the KEGG database. **(C–F)** Comparison of the stability at different taxonomic and functional levels.

To determine the differences in the bacterioplankton communities between the open and closed lakes, we first aimed to determine the taxonomic and functional diversity of the bacterioplankton communities through metagenomic sequencing. A total of 650.07 million high-quality sequence reads with an average length of 126 bp were obtained from 12 sampling sites. A total of 4,487 distinct bacterioplankton taxa were detected across all sampling sites, covering 53 phyla, 1,509 genera and 511 families. The majority of bacterioplankton reads belonged to Cyanobacteria, Proteobacteria and Actinobacteria, accounting for 48.53%, 19.81%, and 19.04% of the total bacterioplankton reads, respectively (Figure 2A). The dominant genera were *Microcystis* (32.58% of reads), *Actinomycetes* (7.29% of reads), *Clavibacter* (5.39% of reads), *Prochlorothrix* (3.51% of reads), and *Acidimicrobium* (3.00% of reads; Figure 2A).

There were 6,695 KEGG orthologues, and 70.77% of the sequences belonged to the metabolism group. In the metabolism group, the “global and overview maps” subgroup accounted for 39.83% of the reads, representing metabolic pathways, biosynthesis of secondary metabolites, amongst others. In addition, amino acid metabolism, carbohydrate metabolism and energy metabolism were the main metabolism types, accounting for 11.00%, 10.70%, and 9.53% of the reads, respectively (Figure 2B).

In general, there were no noteworthy discrepancies observed in the stability of the two lake types at the phylum and functional genes levels (*p* > 0.05), as depicted in Figures 2E,F. There was a significant discrepancy observed at the species and genus levels (*p* < 0.01), with closed lakes exhibiting lower ASV indices and higher stability (Figures 2C,D).

### 3.2. Comparison of taxonomic and functional structures of bacterioplankton communities between open and closed lakes

**Figure 3:**
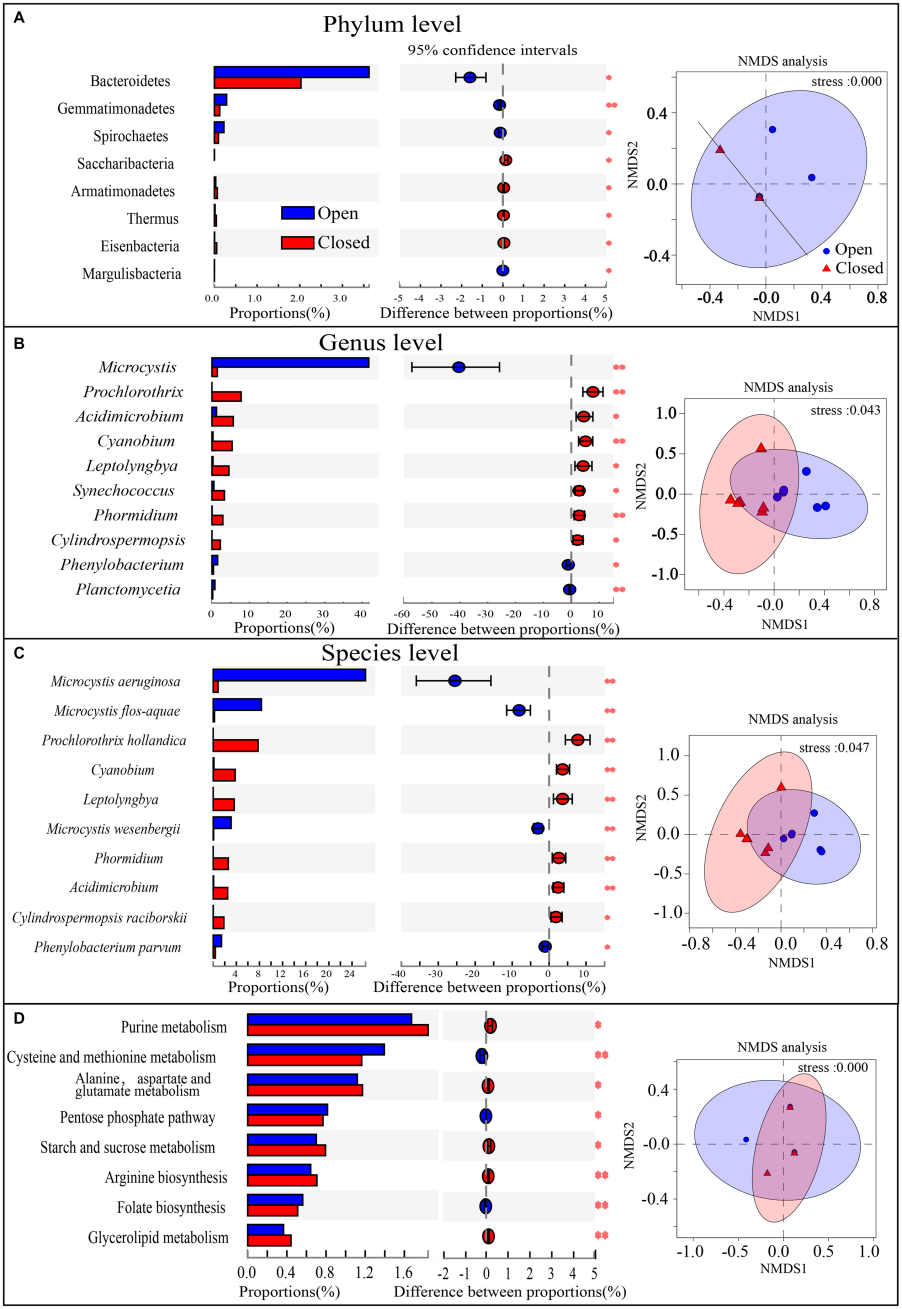
**(A–D)** Species relative abundance or KEGG functional gene relative abundance comparison and significance test between the open lake group and closed lake group, 0.01 < *P* ≤ 0.05 *, 0.001 < *P* ≤ 0.01 **, *P* ≤ 0.001 ***. In addition, NMDS analyses were added, and the 95% confidence intervals are shown.

To compare the community structure differences from the perspective of taxonomy and function, the components with the largest proportion were selected, and their significance was tested. In addition, the similarity pattern of all sample communities was visually displayed by NMDS analyses (Figure 3). The bacterioplankton community structures of the open and closed lakes showed little difference at the phylum level or at a functional gene level (Figures 3A,D) but showed obvious differentiation at the genus and species levels (Figures 3B,C). At the phylum level, the relative abundances of the top 4 phyla, such as Cyanobacteria and Proteobacteria, did not differ significantly between the open and closed lakes (Figure 3A). Only the relative abundance of Bacteroides showed fluctuations, and the NMDS pattern showed that the phylum-level community structures at most sampling stations were very similar (Figure 3A). At the same time, the functional gene patterns were not significantly different between the open and closed lakes, with little difference in the dominant functional genes (Figure 3D).

**Figure 4:**
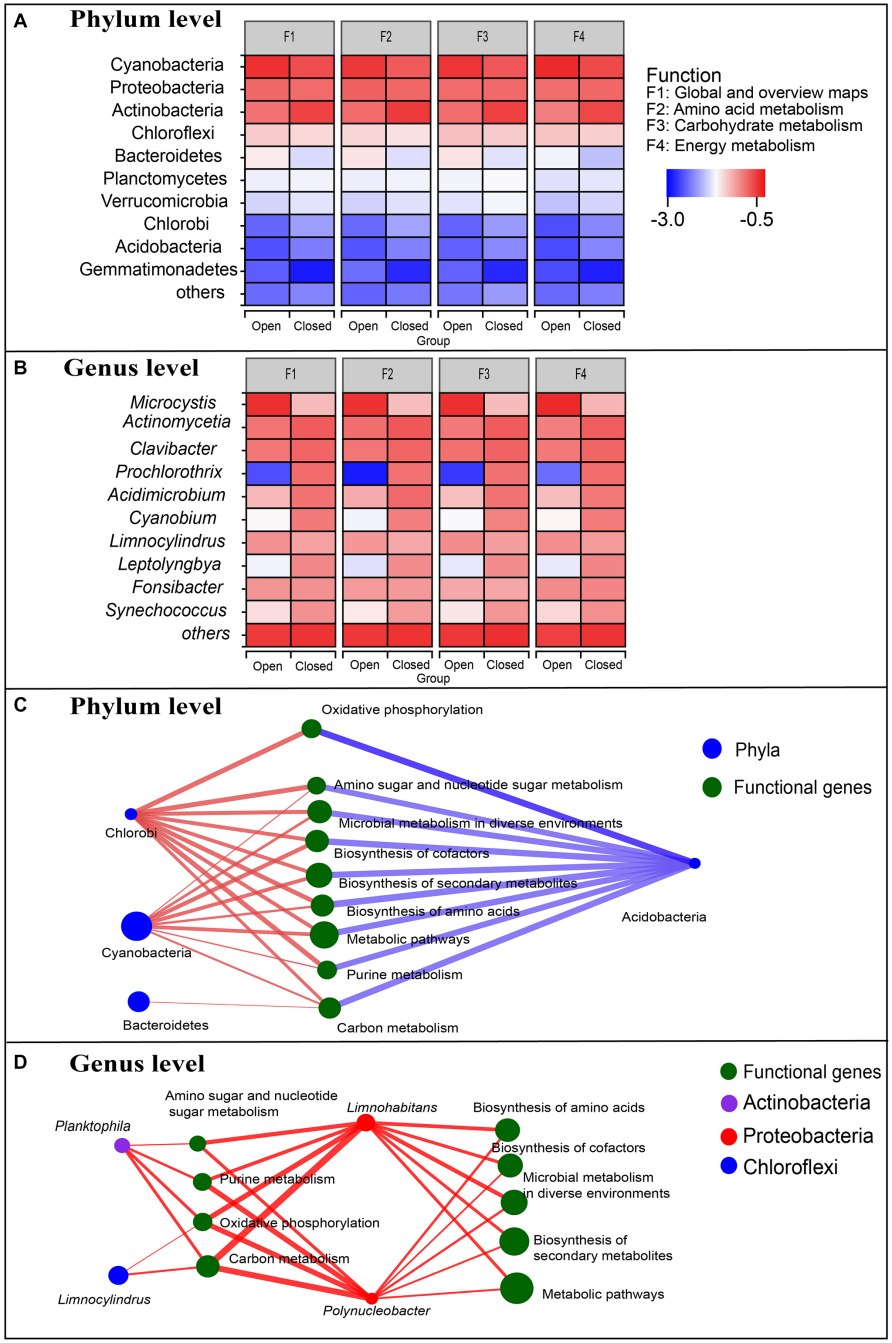
**(A,B)** Comparison of species contributions to metabolic functions between open and closed lakes. Red indicates the highest contribution, and blue indicates the lowest contribution. **(C,D)** Correlation networks between species and functions of the bacterioplankton communities. Larger nodes indicate a higher relative abundance of species or functional genes. The red lines indicate positive and negative correlations, and the blue lines indicate negative correlations.

At the genus level, *Microcystis* was highly abundant in open lakes (proportion = 41.76%) and directly accounted for the differentiation in community taxonomic structures; in contrast, other genera of Cyanobacteria, such as *Prochlorothrix*, *Cyanobium* and *Leptolyngbya*, occurred at significantly higher proportions in the closed lakes (Figure 3B). Furthermore, at the species level, Figure 3C shows that the key cyanobacterial bloom species were *Microcystis aeruginosa*, *Microcystis flos-aquae* and *Microcystis wesenbergii*, whilst *Microcystis aeruginosa* accounted for the primary differences in the communities between the open and closed lakes. In addition, the genus *Acidimicrobium* of Actinobacteria was significantly more abundant (*p* < 0.01) in the closed lakes than in the open lakes (Figure 3B), and *Acidimicrobium* also contributed more to functional genes in the closed lakes than in the open lakes (Figure 4B).

### 3.3. Dominant taxa driving functional differences in bacterioplankton communities

Metagenomic approaches allow structural and functional correlations to be analysed at different levels (Figure 4), reflecting how species composition drives functional composition. At the genus and phylum levels, the species contributions to different functional genes were approximately the same, but there was a significant difference between the open and closed lakes (Figures 4A,B). At the phylum level, the top three phyla with the highest relative abundance (Figure 2A), Cyanobacteria, Proteobacteria and Actinobacteria, were primarily responsible for the metabolic functions of the bacterioplankton communities (Figure 4A). At the genus level, the dominant genera had different responses to the different lake types: *Microcystis*, the most abundant genus, contributed significantly more to the metabolic functions in the open lakes; the contribution of *Prochlorothrix*, *Cyanobium*, and *Leptolyngbya* of Cyanobacteria to metabolic functions decreased significantly in the open lakes and the other genera were not obviously responsive to lake type (Figure 4B).

Figure 4C shows the correlations between the bacterioplankton phyla and metabolic functional genes: Cyanobacteria and Chlorobi were positively correlated with the dominant functional genes (Spearman correlation coefficients > 0.601, *p* < 0.05), and Bacteroidetes were only positively correlated with carbon metabolism (Spearman correlation coefficients =0.587, *p* < 0.05). However, Actinobacteria generally had strong negative associations with these dominant functional genes (Figure 4C). Figure 4D shows the correlations between the dominant genera and dominant functional genes: both *Limnohabitans* and *Polynucleobacter* of Proteobacteria had strong positive associations with the dominant functional genes (Spearman correlation coefficients >0.615, *p* < 0.05), especially carbon metabolism (Spearman correlation coefficients > 0.776, *p* < 0.01) and oxidative phosphorylation (Spearman correlation coefficients > 0.748, *p* < 0.01).

### 3.4. Correlations between bacterioplankton communities and biotic or abiotic factors

**Figure 5:**
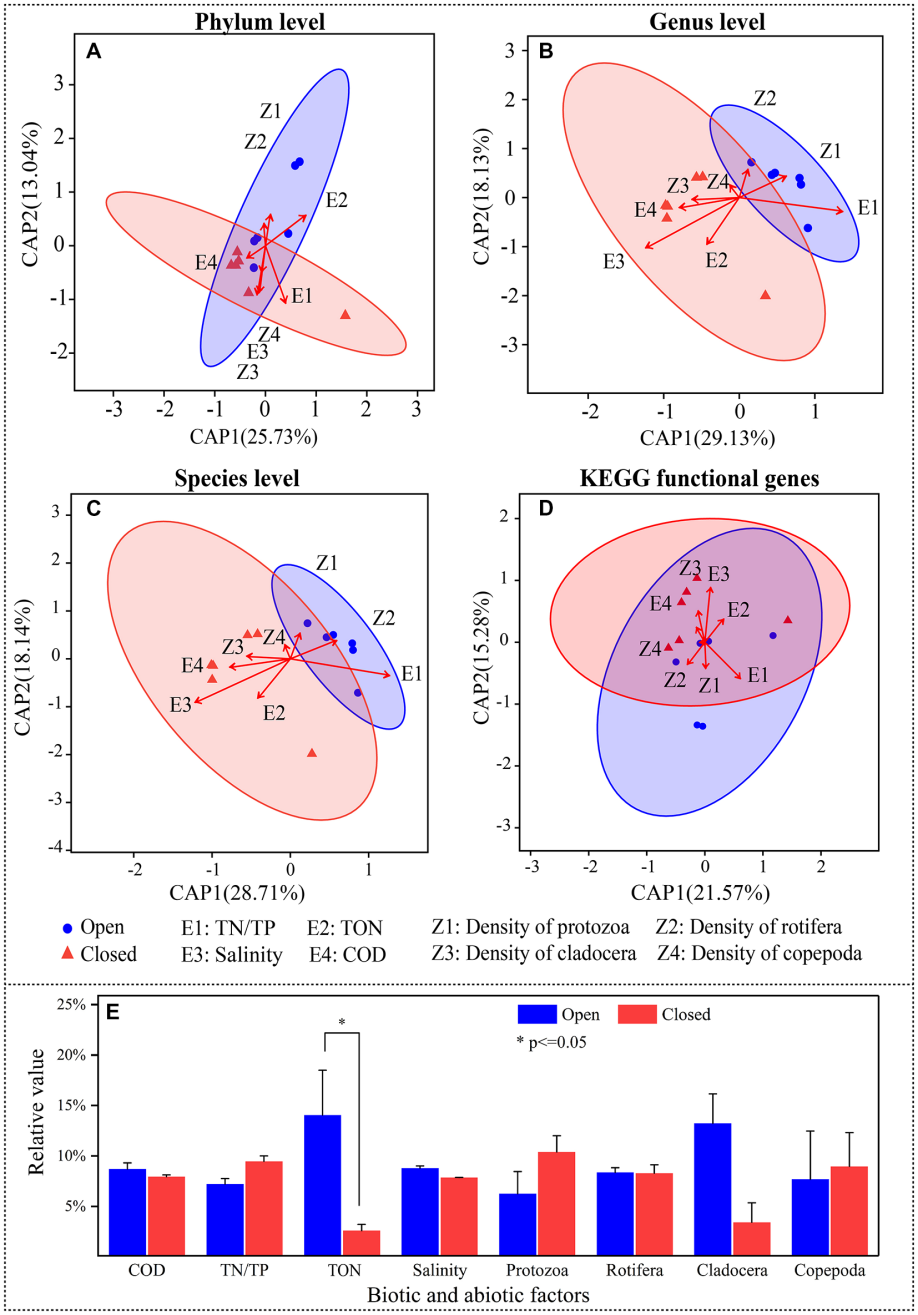
**(A–D)** Distance-based redundancy analysis (db-RDA) with 95% confidence intervals showing the relationships between bacterioplankton species relative abundance or KEGG functional gene relative abundance (response variables) and biotic or abiotic factors (explanatory variables). **(E)** Comparison of biotic or abiotic factors between the open and closed lakes, in which zooplankton densities were selected.

In general, the closed lakes demonstrated significantly lower levels of nitrogen and phosphorus compared to the open lakes, as evidenced by a noticeable reduction across TP, labile P, TN and TON (Figure 5E; Supplementary Figure 2). At the same time, the density of zooplankton did not vary between open and closed lakes.

As mediators of the effects of closed-lake management practices on bacterioplankton, water properties and zooplankton density were used to explain community differences (Figure 5). In the db-RDA models (Figures 5A–C), although the current 8 biotic and abiotic indicators only explained 38.77% to 47.26% of the variation in bacterioplankton community structures, they did reflect the community differences between the open and closed lakes. At different taxonomic levels, there were correlations between zooplankton density and bacterioplankton community structures. For example, Cladocera and Rotifera were correlated with the genera of the bacterioplankton community structure (*R^2^* = 0.539, *p* < 0.05; *R^2^* = 0.503, *p* < 0.05).

On the other hand, the water properties affected the community structures of the bacterioplankton communities (Figures 5A–C). For example, salinity directly affected the bacterioplankton community structure at the species level (*R^2^* = 0.512, *p* < 0.05) and KEGG functional genes (*R^2^* = 0.543, *p* < 0.05). In contrast, although biotic and abiotic indicators can affect the bacterioplankton community taxonomic structures, they did not further significantly affect the functional gene structures (Figure 5D); for example, salinity and Cladocera had no significant effect on the functional gene structures (*R^2^* = 0.275, *p* = 0.239; *R^2^* = 0.493, *p* < 0.05).

## 4. Discussion

Although the relationship between cyanobacterial blooms and the bacterial community has been frequently investigated in previous studies (Chen et al., 2018; Liu et al., 2019), few studies have investigated the taxonomic and functional structures of bacterioplankton communities based on metagenomic sequencing. In this study, to assess the ecological effects of closed-lake management practices, the taxonomic and functional structures of bacterioplankton communities were compared; furthermore, correlations between species and function were revealed and the main taxa that drove community taxonomic and functional structures were identified.

Our study found strong associations between *Microcystis* and 4 major community metabolic functions (Figure 4B), but there was no significant association between *Microcystis* and functional genes (Figure 4D). At the same time, two genera of Proteobacteria accounted for the most abundant functional genes (Figure 4D), and Steffen et al. (2012) also found that metabolic functional genes were mainly related to Proteobacteria in Taihu Lake. Does this suggest that *Polynucleobacter* and *Limnohabitans* of Proteobacteria have a more dominant influence on maintaining the stability of functional gene structure compared to *Microcystis*? The concept of keystone taxa has helped in understanding the changes in community function; keystone taxa determine the main function of a community regardless of their abundance (Banerjee et al., 2018). For example, Chen et al. (2018) found that although *Microcystis* occurred at the highest proportion in the lake, Proteobacteria were associated with metabolic functional genes. Similarly here, the *Microcystis* with the greatest relative abundance had no significant correlation with major functional genes (Figure 4D). Thus, compared with the dominant taxa, keystone taxa may also be a contributor to the relatively conserved functional structure.

On the other hand, Cyanobacteria were dominant in this study, mainly driving aerobic respiration, nitrogen assimilation, nitrogen mineralisation, assimilatory sulphate reduction and other metabolic pathways (Figure 4C), whilst Actinobacteria and Proteobacteria potentially mediated these metabolic processes (Shen et al., 2019). Although Cyanobacteria are not directly involved in many functions, the cyanosphere is critical to the associated heterotrophic bacterial communities. For instance, Shi et al. (2010) stressed that the bacteria in the cyanosphere were likely specifically related to Cyanobacteria. Subsequently, Xie et al. (2016) sequenced a stable community in the laboratory and found a mutually beneficial relationship between the bacteria and *Microcystis*: all heterotrophic bacteria were dependent upon *Microcystis* for carbon and energy, whilst *Microcystis* was dependent upon the heterotrophic bacteria for the vitamin B-12 required for its growth. Furthermore, Smith et al. (2021) isolated single *Microcystis* colonies via droplet encapsulation and found that differences between *Microcystis* strains may impact community composition of the *Microcystis* phycosphere.

In our study, despite significant cyanobacterial blooms in the open lakes, there was no significant difference in cyanobacterial relative abundance between the open and closed lakes (Figure 3A). Specifically, although the relative abundance of *Microcystis* was extremely high in the open lakes, the relative abundance of the other dominant Cyanobacteria genera was significantly reduced (Figure 3B). One possible reason for this result was that the members of Cyanobacteria share a very similar niche (Zhao et al., 2016). Similarly, during a cyanobacterial bloom in the Baltic Sea, Berg et al. (2018) stressed the exceptionally strong biotic driving forces of cyanobacterial blooms on associated microbial communities, but the post-bloom microbial community still re-established a comparable status in terms of diversity to that of the pre-bloom status. In addition, the functional structures of Cyanobacteria did not differ generally between the open and closed lakes (Figure 4A), abundance trade-off may had resulted in a functional structure trade-off in the phylum of Cyanobacteria (Figure 4B). Consistent with these findings of a functional structure trade-off, Steffen et al. (2012) compared the microplanktonic communities amongst Lake Erie (North America), Taihu Lake (China), and Grand Lake St. Marys (United States) and found that despite the variation in the phylogenetic assignments of the bloom-associated organisms, the functional potential remained relatively constant between systems. Our results are consistent with those of previous studies that have found bacterioplankton community structures to be resistant to cyanobacterial blooms.

Unlike Xiao et al. (2019), who found that resource and predator factors play an important role in changes in bacterioplankton communities in Donghu Lake, there were no obvious ecological driving forces of bacterioplankton communities in this study. Specifically, although significantly higher levels of TON were associated with *Microcystis*, the changes in overall community structure were not well explained by biotic and abiotic factors. One possible explanation is that zooplankton have experienced many coevolutionary adaptations; for example, *Daphnia* may feed on *Microcystis*, whilst *Microcystis* may intoxicate *Daphnia* (DeMott et al., 2001; Lemaire et al., 2012), and *Daphnia* have a limited ability to graze on cyanobacterial blooms (DeMott et al., 2001). Furthermore, the hydrological connectivity of the open lake may have played a direct role in the accelerated proliferation of invasive algae (Zhang et al., 2022), thereby impeding or concealing the influential impact of zooplankton.

However, the relationship between zooplankton and cyanobacteria varies widely (Huisman et al., 2018). During a cyanobacterial bloom in open lakes, Jiang et al. (2016) revealed the rapid evolution of tolerance in two cladoceran grazers (*Daphnia pulex* and *Simocephalus vetulus*) to toxic *Microcystis*, and Sadler and von Elert (2014) showed that cladoceran daphnia can successfully inhibit bloom formation. Consistent with this information, Xiao et al. (2019) found that closed-lake management practices resulted in a significant increase in cladoceran species, and cladocerans, as mainly predators, had a significant positive association with bacterioplankton. In general, water quality and zooplankton abundance are not a good explanation for cyanobacterial blooms. On the other hand, a recent study in the Wangyu River based on the ASV structure in eDNA metabarcoding analysis showed that the *Microcystis* in the Wangyu River were different from those in Taihu Lake and the Yangtze River and might act as a potential source of *Microcystis* (Zhang et al., 2022). eDNA metabarcoding can be a powerful tool to reveal habitat-specific biodiversity in lentic systems (Westfall et al., 2020), and the combination of metagenomic sequencing and eDNA metabarcoding can help to more comprehensively reveal the causes of differences in community structure.

## 5. Conclusion

Our findings support the concept that bacterioplankton community structures are resistant to cyanobacterial blooms. In open lakes, *Microcystis* blooms were accompanied by significant reductions in the proportions of several other Cyanobacteria genera, showing a proportional trade-off amongst Cyanobacteria. At the same time, different species contributions to metabolic functions also showed similar trade-offs, which had important implications for higher levels of stability and resistance. On the other hand, both this trade-off and the presence of dominant taxa reflect community stability, suggesting that although closed-lake management practices improve water quality, the focus of success in controlling cyanobacterial blooms is to cut off the biological invasion pathway. Our study further suggests that there are some dominant taxa associated with many important community functions and provides a reference for controlling cyanobacterial blooms and monitoring and managing lakes. This study highlights the importance of closed-lake management practices in controlling cyanobacterial blooms.

## Data availability statement

The original contributions presented in the study are publicly available. This data can be found at: https://www.ncbi.nlm.nih.gov/bioproject; PRJNA948163.

## Author contributions

JC: study design, data and sample collection, sample measurement, and writing—original draft. TZ and YL: assisting sample collection and measurement. LS and DL: assisting sample collection. XL: manuscript revision. SA: study design and manuscript revision. All authors contributed to the article and approved the submitted version.

## Funding

For support, we thank Forestry Science and Technology Innovation and Promotion Project of Jiangsu Province (LYKJ [2022]02, LYKJ-Chanshu [2021]1) and the Fundamental Research Funds for the Central Universities (0211-14380166).

## Conflict of interest

The authors declare that the research was conducted in the absence of any commercial or financial relationships that could be construed as a potential conflict of interest.

## Publisher’s note

All claims expressed in this article are solely those of the authors and do not necessarily represent those of their affiliated organizations, or those of the publisher, the editors and the reviewers. Any product that may be evaluated in this article, or claim that may be made by its manufacturer, is not guaranteed or endorsed by the publisher.
