# Supplementary material for: Abundance trade-offs and dominant taxa maintain the stability of the bacterioplankton community underlying Microcystis blooms
Source: Front Microbiol. 2023 May 19;14:1181341. doi: 10.3389/fmicb.2023.1181341 (PMC10235547; doi:10.3389/fmicb.2023.1181341)
Supplement: Supplementary file 1 [file Data_Sheet_1.PDF]

Supplementary Material

Abundance trade-offs and dominant taxa maintain the stability of the bacterioplankton community underlying *Microcystis* blooms

Jun Chen[1, 2†], Tiange Zhang[1, 2†], Lingyan Sun[1, 2], Yan Liu[1, 2], Dianpeng Li[1, 2], Xin Leng[1, 2],

Shuqing An[1, 2*]

[1] School of Life Science and Institute of Wetland Ecology, Nanjing University, Nanjing 210000, China
[2] Nanjing University Ecology Research Institute of Changshu (NJUecoRICH), Changshu 215500, China
[†] These authors share first authorship.

*** Correspondence:** Shuqing An: anshq@nju.edu.cn

1 Supplementary Figures and Tables

1.1 Supplementary Figures

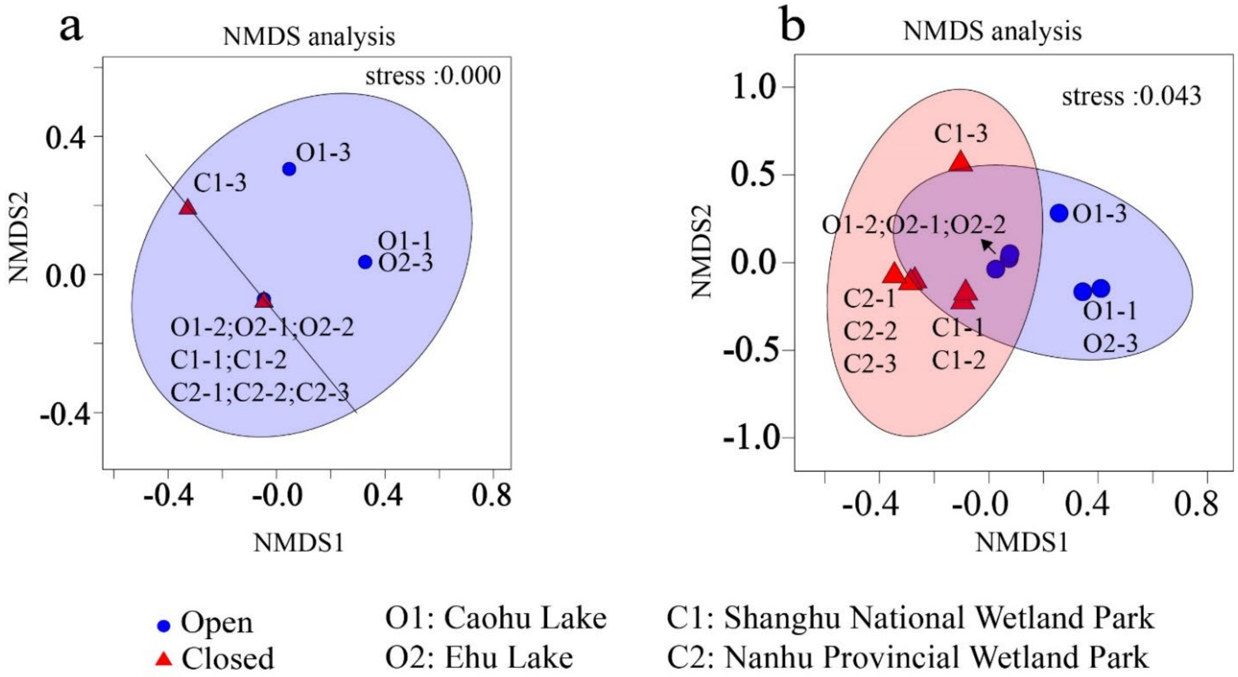

Supplementary Figure 1. NMDS analyses of taxonomic structures and the 95% confidence intervals are shown.

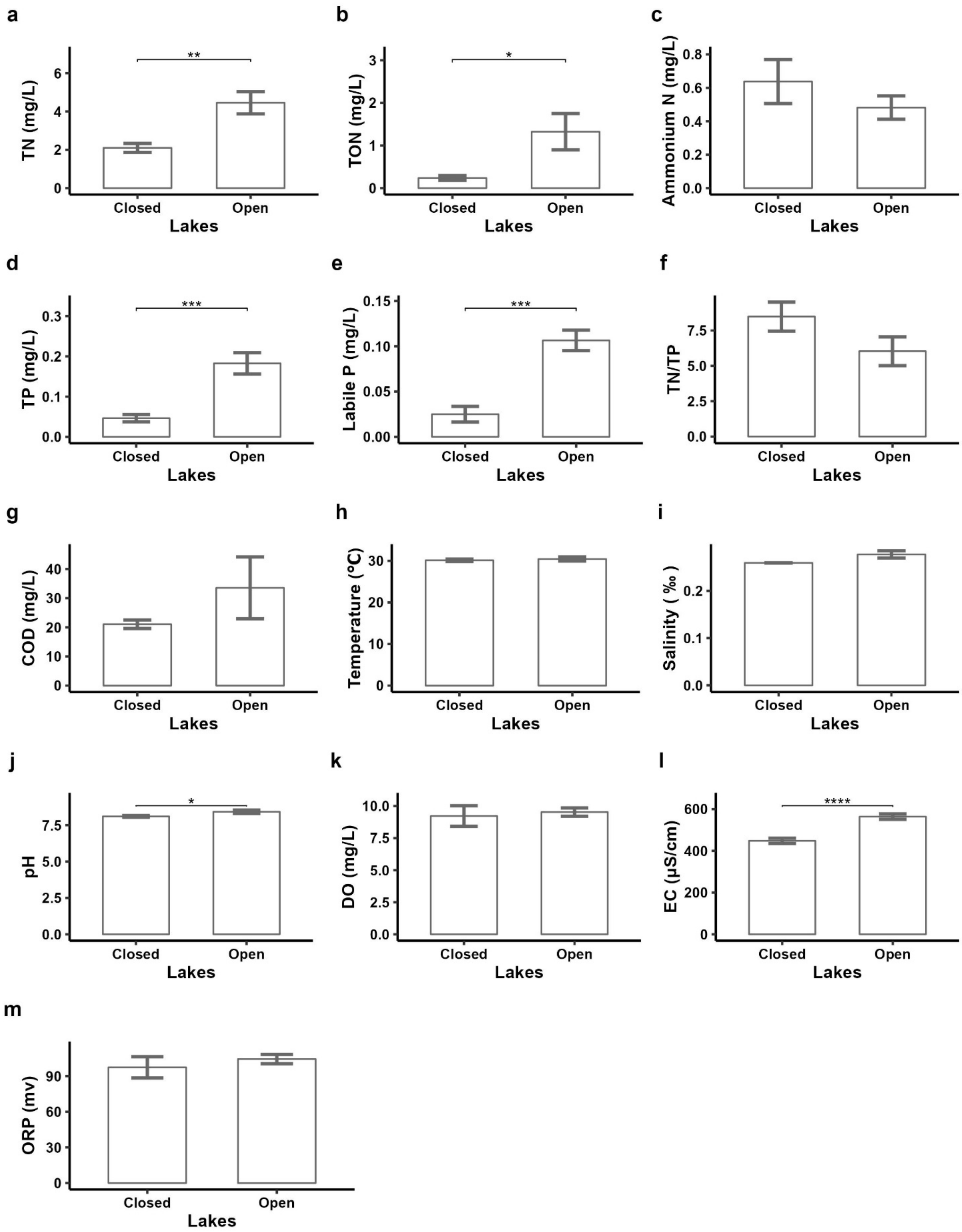

Supplementary Figure 2. The difference in water quality between the open and closed lakes.

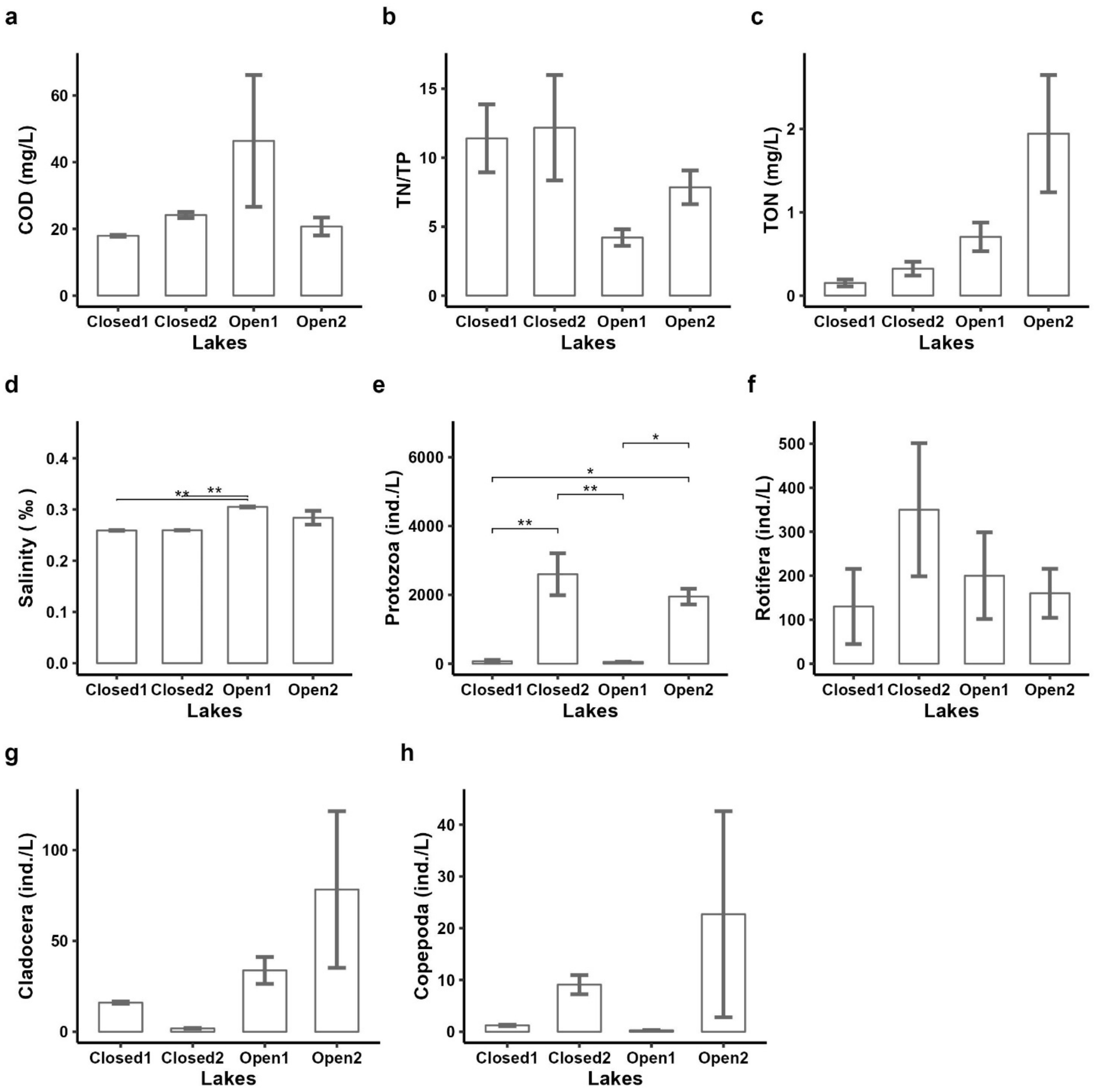

Supplementary Figure 3. Biotic and abiotic factors.
